# Tubular Dysfunction and Ruptured Ureter in a Child with Menkes Syndrome

**DOI:** 10.1155/2021/4398456

**Published:** 2021-08-17

**Authors:** Wun Fung Hui, Kam Lun Hon, Alexander K. C. Leung, Kristine Kit Yi Pang, Michael Wai Yip Leung

**Affiliations:** ^1^Department of Paediatrics and Adolescent Medicine, The Hong Kong Children's Hospital, Kowloon, Hong Kong; ^2^Department of Paediatrics, The University of Calgary and Pediatric Consultant at The Alberta Children's Hospital, T2M 0H5, Calgary, Alberta, Canada; ^3^Department of Surgery, The Hong Kong Children's Hospital, Kowloon, Hong Kong

## Abstract

Children with Menkes disease may develop various urological and renal problems that evolve as the disease progresses. A 4-year-old boy with Menkes disease had multiple bladder diverticula and a history of recurrent urinary tract infection caused by urea-splitting organisms. The child developed urosepsis and right pyelonephritis. Subsequent investigations revealed multiple right renal stones and a ruptured right ureter. The child also developed hypokalemia, hypophosphatemia, and normal anion gap metabolic acidosis that required electrolyte and potassium citrate supplement. Further assessment revealed renal tubular dysfunction. Our case suggests that regular imaging surveillance, monitoring of renal function and electrolyte profile, and tubular function assessment should be considered in children with Menkes disease.

## 1. Introduction

Menkes syndrome, also known as Menkes kinky hair syndrome, is a rare X-linked recessive disease caused by mutation of the *ATP7A* gene. The gene encodes a transmembrane copper-transporting P-type ATPase; mutation of the gene may lead to impaired copper metabolism. Most patients have severe developmental delay, seizures, failure to thrive, and connective tissue abnormality resulting in blood vessel tortuosity and the characteristic kinky hair [1]. Besides, urological abnormalities and related complications such as recurrent urinary tract infection may also be encountered [2, 3]. We report a 4-year-old boy with Menkes disease who presented with urosepsis, and subsequent workup identified a ruptured right ureter resulting from a ureteric stone. The child was also found to have multiple bladder diverticula and tubular dysfunction; the latter led to multiple electrolyte disturbances. Our case illustrated that uncommon urological and renal complications may develop in children with Menkes disease.

## 2. Case History

A 4-year-old boy with Menkes disease was admitted for increased abdominal distension and discomfort for two days associated with reduced oral intake. There was no vomiting, and his urine output and urine appearance were normal. He also developed fever and dyspnea for one day.

During infancy, the child was suspected to have Menkes disease due to fair skin, sparse, kinky, unruly, and steely gray-colored hair, failure to thrive, seizures, and developmental delay. The diagnosis was confirmed at 5 months of age by genetic analysis which showed a *de novo* pathogenic mutation of the *ATP7A* gene. He had been given copper-histidine injection since 8 months of age. He had a gastrostomy with fundoplication performed at 2 years of age and had intractable chronic diarrhea. The child had several episodes of urinary tract infections (UTIs) caused by *Klebsiella* and *Proteus* species since one year of age. An ultrasound of the urinary system at 14 months of age revealed normal kidneys, diffuse urinary bladder wall thickening, and bladder diverticula. There was no ureterocele. The child was found to have a neurogenic bladder with poor bladder emptying, requiring regular intermittent catheterization of the bladder since 19 months of age. The frequency of UTI significantly reduced thereafter, and he was given trimethoprim prophylaxis till 29 months of age. The initial micturating cystourethrogram (MCUG) and DMSA appointments were defaulted. Urodynamic study was unfortunately not performed, and there was no regular imaging surveillance of the urinary system.

Physical examination on admission showed stable vital signs, and his temperature was 36.2°C. The hydration status was satisfactory. The abdomen was distended, and there was generalized tenderness. Investigations showed hemoglobin 8.5 g/dL, white blood cell count 8.97 × 10^9^/L, and platelet count 179 × 10^9^/L. The child was found to have stage 1 acute kidney injury with urea and creatinine levels raised to 11 mmol/L and 47 umol/L, respectively (baseline creatinine level: 25 umol/L). The electrolyte profile showed serum sodium 137 mmol/L, potassium 3.4 mmol/L, chloride 106 mmol/L, calcium 2.74 mmol/L, and phosphate 0.89 mmol/L. There was also normal anion gap metabolic acidosis with venous blood gas showing pH of 7.31, bicarbonate of 14.5 mmol/L, base excess of −10.2 mmol/L, and a calculated anion gap of 16.5 mmol/L. The C-reactive protein was also raised to 212 mg/L. The child was empirically started on intravenous Augmentin after sepsis workup.

**Figure 1:**
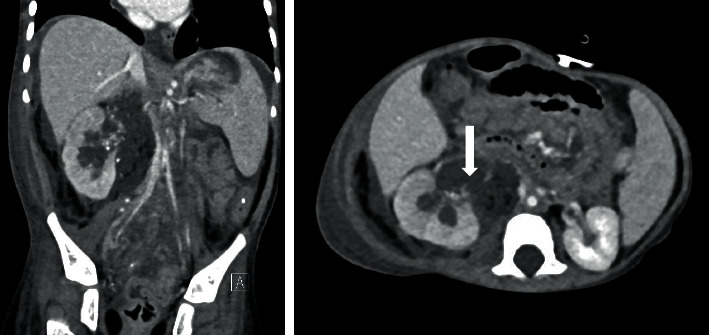
CT image showing rupture of the right ureter. A focal wall defect was noted at the posterior aspect of the right proximal ureter, just distal to the right ureteropelvic junction (arrow) with adjacent fluid surrounding the ureter.

An urgent CT abdomen and pelvis showed diminished right kidney parenchymal enhancement, multiple right kidney and pelvis stones up to 1.5 cm in size, and a right mid-ureteric stone of 0.5 cm with proximal hydroureteronephrosis. A focal wall defect was noted at the posterior aspect of the proximal ureter with contrast extravasation resulting in contrast accumulation around the ureter and in the retroperitoneal space suggestive of a ruptured proximal ureter with urinary leakage (Figure 1). There were multiple bladder diverticula and bladder stones.

The child evolved to develop thrombocytopenia and mildly deranged clotting profile suggestive of disseminated intravascular coagulation. The clinical diagnosis was pyelonephritis and urosepsis complicated with multiple renal stones and ureteric rupture, together with a normal anion gap metabolic acidosis. An emergency operation was then arranged. Intraoperative cystoscopy found stone debris with turbid urine inside the bladder. Multiple bladder diverticula were identified with the left ureteral orifice buried inside a diverticulum, and the right ureteral orifice was not identified. A right percutaneous nephrostomy was performed. The child was then transferred to pediatric intensive care unit for further management.

The urinary drainage was satisfactory after the operation. The blood culture isolated non-typhoidal *Salmonella* Group B. Urinary culture from both the percutaneous nephrostomy, and bladder catheterization identified multiple bacterial species including *Proteus mirabilis*, *Klebsiella pneumoniae*, *Escherichia coli*, *Salmonella* Group B, *Morganella morganii*, and *Streptococcus anginosus*. The antibiotics were later switched to meropenem according to the sensitivity profile.

The patient developed hypokalemia and hypophosphatemia with diuresis requiring electrolyte replacement. The metabolic acidosis also persisted. The acute kidney injury resolved with creatinine returning to baseline levels. Further metabolic and renal tubular function workup was then performed for the renal stone and multiple electrolyte disturbances, and the results revealed tubular dysfunction (Table 1). Because of the active urosepsis, an ammonium chloride loading test was not performed.

He was initially given bicarbonate infusion followed by oral potassium citrate supplement to keep the urine pH > 7.0 and serum bicarbonate level >20 mmol/L. Phosphate supplement was also required to maintain a normal serum phosphate level. Adequate fluid was administered, and a total course of 4-week antibiotics was given. After a thorough discussion, parents agreed for further definitive treatment regarding the urological problem. Right pyeloplasty was then performed 6 weeks later by dividing the ureter proximally at the ureteropelvic junction and distally below the ureteric stricture followed by a ureteropelvic anastomosis, and the stones over the right renal pelvis were also removed. The ureteric stricture that was secondary to ureteric rupture was surrounded by thick fibrotic tissue and attached firmly to the retroperitoneal space. A J-J stent was inserted into the right ureter. Analysis of the right ureteric stone showed 98% carbonate apatite and 2% calcium oxalate monohydrate.

## 3. Discussion

Children with Menkes disease may develop various urological abnormalities, and bladder diverticulum is the most frequently reported one, with a reported prevalence ranging from 36.8% to 57.1% [2, 3]. Lysyl oxidase is a copper-dependent and elastic-fiber-associated coenzyme responsible for lysine-derived cross-linking of collagen and elastin in connective tissue. The function of lysyl oxidase is defective in patients with Menkes syndrome, which leads to connective tissue abnormality including the formation of multiple bladder diverticula [3, 4]. This may lead to neurogenic bladder with urinary stasis, incomplete bladder emptying, and recurrent UTI as illustrated in our patient, which are risk factors for development of pyelonephritis and chronic kidney disease.

Our patient had a ruptured ureter, which is a rare complication of ureteric stone. It may potentially lead to urinoma, retroperitoneal abscess, and urosepsis [5]. Most of the reported cases are associated with ureteric stones [6, 7], and rarely bladder outlet obstruction [8] or connective tissue abnormality [9]. All these risk factors were present in our patient. Hence, the ureteric rupture in our patient could possibly be explained by the formation of urinary tract stones due to recurrent UTI by urea-splitting organisms, together with other predisposing urological or systemic factors.

Given the high incidence of urological abnormalities with their complications in children with Menkes disease, renal system imaging surveillance such as regular ultrasound of the urinary system should be considered to identify and monitor the progression of any urological abnormalities and to detect stone formation. More invasive investigations such as MCUG or cystoscopy may be considered on an individual basis. There is currently no consensus on how to investigate and manage the urological abnormalities among children with Menkes disease. A thorough discussion among the medical team and parents should be encouraged as Menkes disease is considered a life-limiting condition and parents may opt not to perform invasive investigations as in our present case.

Our patient also had tubular dysfunction with pattern of both proximal and distal tubular involvement. Traditionally, urine anion gap is used to differentiate between patients with proximal or distal renal tubular acidosis. However, our patient's chronic diarrhea confounded the results of urinary anion gap, making it difficult for interpretation. In the present case, tubular dysfunction was suggested by the presence of *β*-2-microglobulinuria, generalized aminoaciduria, and urinary electrolyte wasting. Tubular dysfunction in Menkes disease is not well studied compared to Wilson's disease, another disorder of copper metabolism. Previous reports showed conflicting results regarding the tubular function assessment in patients with Menkes disease [10, 11]. Urinary *β*-2-microglobulin level has been used as a marker for copper-histidine therapy-associated proximal tubular dysfunction among children with Menkes disease [12]. Although copper deposition has been demonstrated in the proximal renal tubules among patients with Menkes disease receiving copper-histidine treatment [13], there were also reports describing tubular dysfunction in children not given copper-histidine therapy [10]. Ozawa et al. reported the results of serial renal function assessment on three patients with Menkes disease that were given copper-histidine therapy, and one of them showed an elevated pretreatment urinary *β*-2-microglobulin level [10]. Interestingly, the urinary *β*-2-microglobulin level increased in all three of them as the patient grew older. It is still not certain whether tubular dysfunction is purely a clinical manifestation of children with Menkes disease or a consequence of copper-histidine therapy. Currently, there are no longitudinal data on the evolution of tubular dysfunction among children with Menkes disease and its relation to the administration of copper-histidine therapy. However, the results of our patient suggest that regular monitoring of renal function and electrolyte profile with tubular function assessment may be required in children diagnosed with Menkes disease, especially for those who have been given copper-histidine therapy.

## 4. Conclusion

Menkes disease is a multisystem disorder caused by defective copper metabolism. Urological abnormality is not uncommon, which may lead to rare complications such as a ruptured ureter. In addition, tubular dysfunction may also be encountered leading to electrolyte and acid-base disturbances. Hence, regular imaging surveillance of the urinary system, monitoring of renal function and electrolyte profile, and renal tubular function assessment may be needed in these patients.

## Figures and Tables

**Table 1:** Urinary indices of tubular function assessment.

Urinary indices	Value
Urine pH	6.5
Urine anion gap	10
Urine beta-2-microglobulin (ug/ml) (normal: <0.2 ug/ml)	65.1
Aminoaciduria (%)^*∗*^	100%
Urine potassium-creatine ratio (mmol/mmol Cr)	18.0
Transtubular potassium gradient	8.0
24-hour urinary calcium (mmol/kg/day) (renal wasting: >0.1 mmol/kg/day)	0.15
Tubular maximum phosphate reabsorption (renal wasting: <1.15)	0.38
Tubular reabsorption of phosphate (%) (renal wasting: <85%)	24.4%
24-hour urinary magnesium (mmol/day) (renal wasting: >1 mmol/day)	0.28
24-hour urinary uric acid (mmol/day) (normal: 1.2–5.9)	1.0

^*∗*^Expressed as percentage of types of amino acids with measured values exceeding the upper limit of normal range. Tubular dysfunction was suggested by the presence of *β*-2-microglobulinuria, generalized aminoaciduria, and urinary electrolyte wasting. The chronic diarrhea in our patient confounded the interpretation of urine anion gap.

## Data Availability

No data were available.
